# THE MEANING OF EXPERIENCING FEAR OF FALLING AMONG OLDER ADULTS

**DOI:** 10.1093/geroni/igac059.3094

**Published:** 2022-12-20

**Authors:** Hanne Dolan, Natalie Pool

**Affiliations:** Arizona State University, Phoenix, Arizona, United States; University of Northern Colorado, Greeley, Colorado, United States

## Abstract

Fear of falling (FOF) is prevalent among older adults. While the concept has been conceptually defined and factors associated with FOF has been extensively explored in nursing and other health sciences, the experience of this fear is often overlooked. The purpose of this study was to illuminate the phenomena of FOF using the rich descriptions elicited from four (Nf4) older adults. The meaning of these experiences was then interpreted in conjunction with the known and emergent FOF body of literature. Each participant was interviewed twice using videoconferencing as part of a larger study exploring the lived experience of being at risk for falling in the hospital. A total of eight interview transcripts were analyzed using van Manen’s interpretive phenomenological methodology. The philosophical underpinning of this study is the philosophy of caring in nursing outlined by Kari Martinsen. Three major interpretive themes emerged: Loss of Self, Part of my Existence, and Remaining Safe Within the Boundaries of Fear. These themes describe how FOF is the fear of being suspended in time and space and losing connection with oneself both physically and mentally during a fall. The fear becomes a part of one’s existence, ranging from worry to all-consuming panic, and the body becomes unpredictable. FOF means living within invisible boundaries of fear, where feelings of helplessness, uselessness, and isolation are common. Relationships with others can both temper and ignite the FOF, and caregivers must understand the meaning of this experience to improve support of older adults in managing this overwhelming experience.

